# Maxillary Sinus Assessment: A Computed Tomography Analysis and Classification

**DOI:** 10.1055/s-0044-1791728

**Published:** 2025-04-15

**Authors:** Mohammad Waheed El-Anwar, Mohamed Kamel Alawady, Hoda Ismail Abdelhamid, Tamer Oraby, Mohamed Talaat Albasiouny, Ashraf El-Hussiny

**Affiliations:** 1 Department of Otorhinolaryngology, Head and Neck Surgery, Faculty of Medicine, Zagazig University, Zagazig, Egypt; 2 Department of Otorhinolaryngology, Faculty of Medicine, Al-Azhar University, Egypt; 3 Department of Otorhinolaryngology, Ain Shams University, Cairo, Egypt

**Keywords:** maxillary sinus, CT, orbital floor, nose, endoscopic sinus surgery

## Abstract

**Introduction** The preoperative assessment of the computed tomography (CT) characteristics of the maxillary sinus helps to preserve its anatomical and functional integrity during and after surgery.

**Objective** To use CT scanning to identify maxillary sinus variations and types that were not previously published.

**Methods** The present study was carried out on 110 paranasal CT scans (220 sides). Axial images were obtained with multiplanar scans, to visualize details in coronal and sagittal planes for all subjects.

**Results** Among the 110 CTs (220 sides) of the maxillary sinus's floor, there were 53.2% type 1, 29.1% type 2, 10% type 3, and 7.7% type 4, with significant difference between genders. The most common maxillary sinus floor was type 1. The lateral maxillary sinus wall was found to be type 1 in 32.7%, type 2 in 65%, and type 3 in 2.3%, with a significant difference between genders. The most common lateral wall of the maxillary sinus type was type 2. The orbital floor was found to be type 1 in 0.9%, type 2 in 21.3%, type 3 in 50.5%, and type 4 in 27.3%, without significant difference between genders. Asymmetry was detected between the right and left sides for the maxillary sinus floor of in 22.7%, lateral maxillary wall in 16%, and orbital floor (maxillary roof) in 30%.

**Conclusion** This study aims to increase surgeons' awareness of maxillary sinus variations, creating new classifications for usage and communication in the otorhinolaryngology and endoscopic fields. It could also be helpful for training medical residents.

## Introduction

The maxillary sinus is the largest paranasal sinus, being considered as a complex structure with inner- and intra- individual variations 1 2 and with respectable variations in shape and size between the left and right sides. 3 4 5 6 This structure reaches a mature size at the age of 20 years, after full development of permanent teeth. Thus, development is related to changes in the pneumatization extension, 7 and consequently sinus shape and size. 8

The maxillary sinus has an important anatomical structure in different interventions in rhinology, ophthalmology, neurosurgery, and maxillofacial surgery. The preoperative assessment of the pneumatization characteristics helps to preserve its functional and anatomical integrity. Also, a detailed clinical understanding of the maxillary sinus anatomy and variations is important to improve recent surgical techniques and prevent surgical complications. 9

Today, endoscopic sinonasal surgery (ESS) is one of the most frequent in otorhinolaryngology. 10 11 With the evolution in sinoscope technology, tools, and imaging modalities, ESS became more popular. Proper imaging detail is a tool that could be in use to get an effective and safe ESS. Computed tomography (CT) scans are of great importance to evaluate sinonasal disease and to identify the anatomic variations of the sinuses and their near areas, 11 12 13 considering they can significantly differ even between sides in the same person. 13

Therefore, the ESS surgeons should have the detailed preoperative assessment of maxillary sinus and its relation to the orbital and nasal floors. Such detailed knowledge of the sinus's anatomy is critical for complete, safe, and effective ESS. 1 2 The orbit represents an important nearby structure during endoscopic maxillary sinus work, so their relation must be well known.

Knowledge of maxillary sinus variations could reduce complications, disruptions, and/or injuries during ESS. Thus, preoperative MSCT with detailed assessment of this structure is essential.

Even though the maxillary sinus was studied before, 3 4 5 there are limited literature works available about the different variations and classification in CT studies, with no data standardization.

Additionally, the CT details of the maxillary sinus variations and types are not fully covered in the literature, so it is important to create a base for description and categorization of that area. Additionally, preoperative details of are essential to any approach or procedure involving or crossing this structure.

Thus, the objective of this study was to detect descriptions, variations, and grades of the maxillary sinus that were not published before. Our results might influence surgical interventions for the maxillary sinus, particularly the ESS.

## Methods

This cross-sectional analysis was conducted on 110 paranasal sinuses CTs (220 sides) at the otorhinolaryngology department in a University Hospital, between November 2022 and 2023. To participate in the study, all patients signed an informed consent after discussion of its purpose. An institutional review board (IRB) approved the study.

The study followed the declaration of Helsinki's guidelines on Biomedical Research for Human Subjects. Patients < 18 years old, with history of trauma, sinonasal surgery, or with neoplasms, congenital anomaly, and/or sinonasal fibro-osseous lesions were excluded from this study.

Radiologic evaluation via CT was performed with a 64-slices MDCT (Multi detector computed tomography) scans (Light speed volume VCT - GE medical system, Milwaukee, WI, USA) using 1.5mm width sections, a 0.625mm width detector, and 0.5mm interval reconstruction.

For the paranasal sinuses, axial cuts were done taking the beam parallel to the hard palate, with the subjects in a supine position, starting from the hard palate to the frontal sinuses (glabela), utilizing 130 KV and 150 mA/s, with a 1.5s scan time. Scans were done with a bone window setting of 3,000 HU, centered at 700 HU. A high-resolution algorithm was used to improve quality of the delicate bone detail.

**Fig. 1:**
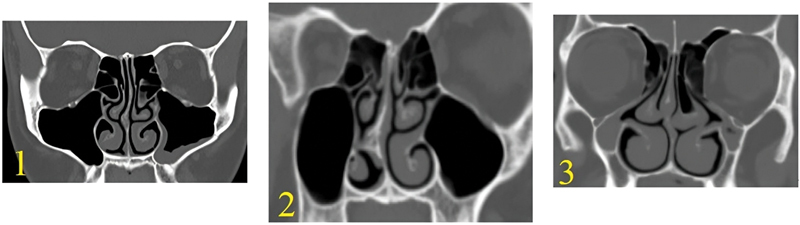
A CT scan of the different types of the lateral wall of the maxillary sinus.

Multiplanar reconstruction with delicate details of the coronal planes were acquired at a dedicated postprocessing workstation (Advantage Windows Volume share 4.5, GE Medical System, Milwaukee, WI, USA). All scans were reviewed in a regular, standardized manner. The maxillary sinus was evaluated along the coronal plane ( Figs. 1 2 3 ).

**Fig. 2:**
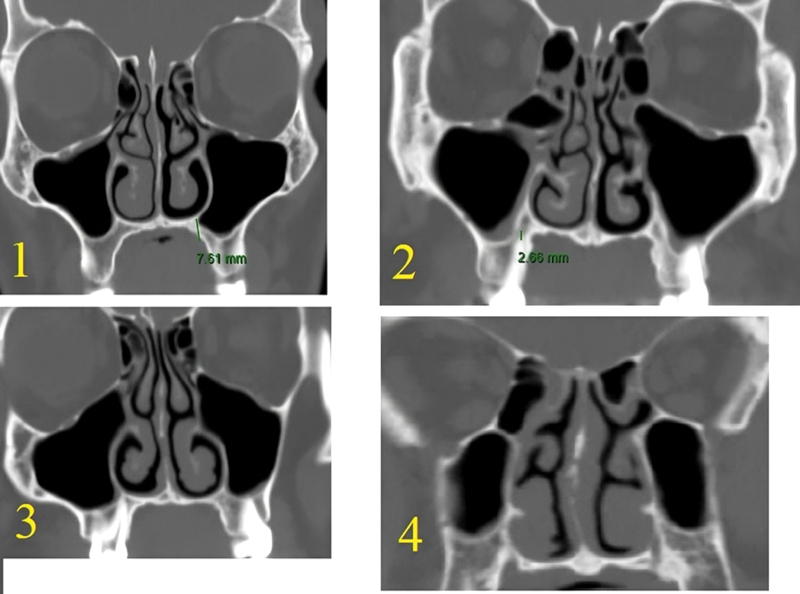
A CT scan of the different grades of the maxillary sinus floor.

**Fig. 3:**
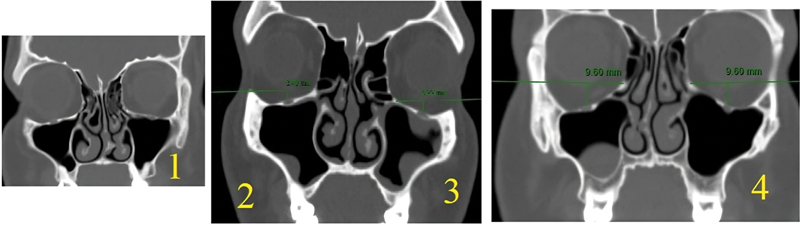
A CT scan of the different grades of the maxillary sinus roof.

The maxillary sinus's floor was categorized into 4 types (grades): 1–below the nasal floor by ≥ 0.5 cm; 2–below the floor by < 0.5 cm; 3–at floor; and 4–above nasal floor ( Fig. 2 ). Regarding the maxillary sinus wall, for type 1 it was at or lateral to the lateral orbital wall; for type 2, it was between the lateral orbital wall and midpoint of the orbital floor; and for type 3, it was between the midpoint of the orbital floor and the medial orbital wall ( Fig. 1 ).

The maxillary sinus's roof represents the floor of the orbit. It was evaluated at the maxillary osteoma level and categorized into: type 1 at the medial orbital ridge level; type 2 below the medial orbital ridge level by ≤ 4 mm; type 3 below the medial orbital ridge by 4 to 8 mm; and type 4 below the medial orbital ridge by ≥ 8 mm ( Fig. 3 ).

Statistical analysis was done with the IBM SPSS Statistics for Windows (IBM Corp., Armonk, NY, USA) version 25. We considered *p* -values < 0.05 as statistically significant.

## Results

The present study involved 110 CTs (220 sides), of which 72 were from males (65.5%) and 38 females (34.5%). The mean age was 34.5 ± 10.4 (20–80 years) years. The mean age was 34.3 ± 8.3 years in females and 34.7 ± 11.3 years in males, with no significant difference ( *p* = 0.8099).

The maxillary sinus floor was found to be type 1 in 117 (53.2%), type 2 in 64 (29.1%), type 3 in 22 (10%), and type 4 in 17 (7.7%). In females (n = 76), type 1 was found in 30 (39.5%), type 2 in 29 (38.2%), type 3 in 11 (14.5%), and type 4 in 6 (7.8%). In males (n =144), type 1 was found in 87 sides (60.5%), type 2 in 35 (24.3%), type 3 in 11 (7.6%), and type 4 in 11 (7.6%), with a significant difference between both gender (X ^2^ = 12.268, *p* = 0.0065) ( Table 1 ). The most common maxillary sinus floor type was found to be type 1 (at a level below the nasal floor by ≥ 0.5 cm) in males and females.

**Table 1:** Comparison between females and males

		Males (72 patients, 144 sides)	Females (38 patients, 76 sides)	*P* -value
**Age**	**Mean**	34.7	34.2	0.8099 NS (t = 0.2411)
	**SD**	11.3	8.3	
**Floor of the maxillary sinus**	**Type 1**	87 (60.5%)	30 (39.5%)	0.0065 (X ^2 ^ = 12.268
	**Type 2**	35 (24.3%)	29 (38.2%)	
	**Type 3**	11 (7.6%	11 (14.5%)	
	**Type 4**	11 (7.6%)	6 (7.8%)	
**Lateral wall of maxillary sinus**	**Type 1**	44 9 (30.6%)	28 (36.8%)	0.0086 (X ^2 ^ = 9.505)
	**Type 2**	97 (67.4)	46 (60.6%)	
	**Type 3**	3 (2%)	8 (2.6%)	
**Floor of orbit (roof of maxillary sinus)**	**Type 1**	1 (0.7%)	1 (1.3%)	0.78 (X ^2 ^ = 0.78)
	**Type 2**	30 (20.9%)	17 (22.4%)	
	**Type 3**	76 (52.8%)	35 (46.1%)	
	**Type 4**	37 (25.7%)	23 (30.2%)	

The lateral wall of the maxillary sinus was found to be type 1 in 72 (32.7%), type 2 in 143 (65%), and type 3 in 5 (2.3%). In females, type 1 was found in 28 (36.8%), type 2 in 46 (60.6%), and type 3 in 8 (2.6%); while in males, type 1 was found in 44 (30.6%), type 2 in 97 (67.4%), and type 3 in 3 (2%), with significant difference between both gender ( *t* = 9.505, X ^2^ = 0.0086) ( Table 1 ). So, the most common lateral wall of the maxillary sinus type was found to be type 2 (Between lateral wall of the orbit and midpoint of the orbital floor) in males and females.

The floor of orbit was found to be type 1 in 2 (0.9%), type 2 in 47 (21.3%), type 3 in 111 (50.5%), type 4 in 60 (27.3%). In females; type 1 in 1 (1.3%), type 2 in 17 (22.4%), type 3 in 35 (46.1%), type 4 in 23/76 (30.2%), and in males, type 1 in 1/144 (0.7%), type 2 in 30 (20.8%), type 3 in 76 (52.8%), type 4 in 37 (25.7%) without significant difference between both sides ( *p* = 0.78, X ^2^ = 1.093). The most common orbital floor was type 3 (below medial orbital ridge by 4–8 mm) in regardless of gender.

Asymmetry was detected between the left and right sides of the maxillary sinus floor in 25 (22.7%), the lateral maxillary wall in 18 (16%), and the floor of the orbit (maxillary roof) in 33 (30%). All asymmetry by one grade only, except for 1 patient (0.9%) with an asymmetry of 2-grade difference on the floor of the maxillary sinus.

## Discussion

Most paranasal sinuses drain within the osteomeatal area, so ESS usually targets the middle meatal area and the maxillary sinus. 6 The regular diagnostic and preoperative evaluation tool for the maxillary sinus anatomy and diseases is CT, with its use guiding ESS, maxillofacial and external sinonasal procedures. Therefore, radiologists and surgeons need know the CT details of the maxillary sinuses to do a safe and successful operation. 4 Maxillary sinus pneumatization varies considerably between genders, ethnic groups, etc., 7 and its anatomy could show differences even between both sides of a same subject. 1 2 Differences in the degrees of maxillary sinus pneumatization are related to variations in its bony walls including the lateral maxillary wall, maxillary roof (orbital floor), and maxillary sinus floor (D1). 14

In the current study, we included subjects older than 20 years, when the maxillary sinus develops its mature size after full development of the permanent teeth. 8 We described anatomical variations in the walls of maxillary sinus: lateral wall, orbital floor (maxillary roof), and floor of maxillary sinus. A new classification (grades, types) for the lateral maxillary sinus extension was used, reporting that type 2 was the most frequent, in which it extends laterally to a level between the lateral wall of the orbit and midpoint of the orbital floor. Additionally, we generated the orbital floor level (roof of the maxillary sinus) types, recording that the most frequent orbital floor was type 2 (reaching below the level of medial orbital ridge by more than 4 mm). These reflect the value of angled endoscopy, bone removal for the maxillary sinus' endoscopic approaches, and choices between approaches to the sinus or crossing it.

It was reported that the different maxillary types showed wide variations, which highlights the importance of studying the CTs to identify each maxillary sinus's type preoperatively, allowing physicians to choose the best approach and instruments for each patient.

Increasing types of the maxillary sinus (types 3 and 4) in our classifications of each wall could represent a surgical challenge and a risk factor for orbital complication when identifying the maxillary sinus osteoma and its opening, maxillary sinus clearance, and others. It could also be a risk factor for disease growth beyond the sinus, with worse prognosis, and may need more care during surgery. Studying of these types of the maxillary sinus in difference maxillary sinus pathology and diseases is suggested.

The present study puts a basic knowledge for the CT detailed description of the maxillary sinus classifications and updates orientations about the maxillary sinus from a CT perspective to rase radiologists' and surgeons' knowledge for more effective and safer ESS, improvement of surgical plans, and preparations for the necessary instruments set in each case.

We could not compare our results and classifications with previous studies, as these data are missing in the literature. Thus, studying the presented new maxillary sinus types of are recommended in in different ethnic groups and various maxillary sinus pathology.

Lerno 15 categorized the maxillary sinus shapes into triangular, rectangular, oval, curved, and square, while the base's shapes were categorized into triangular, leaf, renal, and scapular. However, we think that such classifications do not add to the ESS surgeon. In the current study, asymmetry between both sides of the maxillary sinus was detected in up to 30% of cases, with differences in asymmetry between each wall of the sinus reflecting the importance of revising the type of each wall preoperatively. This result is in agreement with the findings of Shahbazian et al., 16 who noted 17% sinus asymmetry and 83% symmetry. However, Amusa et al. 17 registered 100% sinuses asymmetry in 24 human dried skulls from Southwestern Nigeria.

None of authors described variations in anatomy of lateral maxillary wall, orbital floor (maxillary roof) but most of them described maxillary floor and its relation to teeth development that is important in orthognathic surgery, we are the first authors to describe variations in anatomy of the lateral maxillary wall and orbital floor (maxillary roof), which are important in ESS.

The large maxillary sinus that has deep low floor > 0.5 cm below nasal floor (type 1) has challenges during ESS because the access to the deep floor through maxillary ostium by 0 or 30 degrees could be difficult, and a 70 degree endoscope may be needed to see and remove residual pathology in the deep sinus floor with taking in consideration during surgery, we may need to approaches rather than middle meatal antrostomy like prelacrimal approach or partial resection of the superior border of the inferior turbinate to remove residual pathology in the deep sinus floor.

Therefore, we agree with Sadeghi et al. 18 and Hildenbrand et al., 19 who mentioned that by the conventional rigid endoscopy, medial antrostomy may be not sufficient to visualize and reach all types of maxillary sinus pathologies particularly in diseases on the anterior, anterolateral or inferior maxillary sinus parts. In these specific cases, more invasive approaches with wider accesses are required. For example, the inferior turbinate and/or the nasolacrimal canal could be resected, 18 or a prelacrimal approach can be chosen instead. 19 Alternatively, a canine fossa aperture may be created (Caldwell–Luc procedure). 20

Also, we agree with Lee et al. 21 that in a smaller sinus (type 3 or 4 in our classification), as in the Zulu population, the middle meatus will be more lateral and inferior, so the path to the meatus becomes more oblique and, thus, more complex, meaning the orbital wall will be more at risk for surgical damage.

On the other hand, Wagner et al. 22 measured the sinus depth, from the hard palate to the deepest sinus point, and noted types 1 (above 25%), 2 (0–6 mm below, 50%), and 3 (> 6 mm below, 25%) in CT scans. They concluded that sinus depth is a dependable landmark noted that the access to the maxillary sinus floor through the inferior meatus, for inferior intranasal antrostomy or sinus washout procedures, will be more restricted in type III. It should be noted, however, that inferior intranasal antrostomy is now considered obsolete.

The use of CT is highly valuable to evaluate the maxillary sinus. However, it is worth noting that previous studies have shown a nonstandardized methodology that may have a direct effect on the data discrepancy concerning the maxillary sinus. Thus, the use of classifications as in the current study could be an important step in data standardization during revising, discussing, and transferring the CT of the maxillary sinus between the otorhinolaryngologists, orthognathic surgeons, and radiologists.

To avoid surgical complications, it is critical to include the maxillary sinus's presented types in the review of preoperative CT when planning ESS and dental implant procedures. Further studies should handle the effect of these maxillary sinus variations on related pathologies and surgeries.

## Conclusion

The present study seeks to update the CT awareness about the maxillary sinus to increase radiologists' and surgeons' knowledge of maxillary sinus for more effective and safer ESS. New classifications of the maxillary sinuses were presented, which might offer some useful data in the comprehension of the maxillary sinus's anatomy and surgical applications.

## References

[1] Kojima K Endo T Shimooka S Effects of maxillary second molar extraction on dentofacial morphology before and after anterior open-bite treatment: a cephalometric study Odontology 2009 97 01 43 50 19184297 10.1007/s10266-008-0093-0

[2] Uthman A T Al-Rawi N H Al-Naaimi A S Al-Timimi J F Evaluation of maxillary sinus dimensions in gender determination using helical CT scanning J Forensic Sci 2011 56 02 403 408 21210803 10.1111/j.1556-4029.2010.01642.x

[3] McDonnell D Esposito M Todd M E A teaching model to illustrate the variation in size and shape of the maxillary sinus J Anat 1992 181(Pt 2):377 380 1295877 PMC1259734

[4] William P L Bannister L H Berry M M Collins P Dyson M Dussek J E Gray's Anatomy, (38th Edition), Churchill Livingstone,Edingburgh1995 295 302

[5] Sinnatamby C S, Ed.Last's Anatomy. Regional and Applied (11th ed.) Churchill Livingstone,Edinburgh2005 386 390

[6] Emirzeoglu M Sahin B Bilgic S Celebi M Uzun A Volumetric evaluation of the paranasal sinuses in normal subjects using computer tomography images: a stereological study Auris Nasus Larynx 2007 34 02 191 195 17084569 10.1016/j.anl.2006.09.003

[7] Smith T D Siegel M I Mooney M P Burrows A M Todhunter J S Formation and enlargement of the paranasal sinuses in normal and cleft lip and palate human fetuses Cleft Palate Craniofac J 1997 34 06 483 489 9431465 10.1597/1545-1569_1997_034_0483_faeotp_2.3.co_2

[8] Jovanović S Jelicić N Kargovska-Klisarova A [Postnatal development and anatomical relationship of the maxillary sinus] Acta Anat (Basel) 1984 118 02 122 128 6702412

[9] Timmenga N M Raghoebar G M Liem R S van Weissenbruch R Manson W L Vissink A Effects of maxillary sinus floor elevation surgery on maxillary sinus physiology Eur J Oral Sci 2003 111 03 189 197 12786948 10.1034/j.1600-0722.2003.00012.x

[10] El-Anwar M W Khazbak A O Eldib D B Algazzar H Y Anterior ethmoidal artery: a computed tomography analysis and new classifications J Neurol Surg B Skull Base 2021 82(3, Suppl 3)e259 e267 34306947 10.1055/s-0039-3400225 PMC8289549

[11] Bayram M Sirikci A Bayazit Y A Important anatomic variations of the sinonasal anatomy in light of endoscopic surgery: a pictorial review Eur Radiol 2001 11 10 1991 1997 11702133 10.1007/s003300100858

[12] El-Anwar M W Khazbak A O Hussein A Saber S Bessar A A Eldib D B Sphenopalatine foramen computed tomography landmarks J Craniofac Surg 2020 31 01 210 213 31469730 10.1097/SCS.0000000000005857

[13] El-Anwar M W Khazbak A O Eldib D B Algazzar H Y Lamina papyracea position in patients with nasal polypi: A computed tomography analysis Auris Nasus Larynx 2018 45 03 487 491 28942101 10.1016/j.anl.2017.09.006

[14] Ariji Y Ariji E Yoshiura K Kanda S Computed tomographic indices for maxillary sinus size in comparison with the sinus volume Dentomaxillofac Radiol 1996 25 01 19 24 9084281 10.1259/dmfr.25.1.9084281

[15] Lerno P Identification par le sinus maxillaire Odontol Leg 1983 216 39 40 6581939

[16] Shahbazian M Xue D Hu Y van Cleynenbreugel J Jacobs R Spiral computed tomography based maxillary sinus imaging in relation to tooth loss, implant placement and potential grafting procedure J Oral Maxillofac Res 2010 1 01 e7 10.5037/jomr.2010.1107 PMC3886045 24421963

[17] Amusa Y B Eziyi J AE Akinlade O Volumetric measurements and anatomical variants of paranasal sinuses of Africans (Nigerians) using dry crania Int J Med Sci 2011 3 299 303

[18] Sadeghi N Al-Dhahri S Manoukian J J Transnasal endoscopic medial maxillectomy for inverting papilloma Laryngoscope 2003 113 04 749 753 12671441 10.1097/00005537-200304000-00031

[19] Hildenbrand T Weber R Mertens J Stuck B A Hoch S Giotakis E Surgery of inverted papilloma of the maxillary sinus via translacrimal approach-long-term outcome and literature review J Clin Med 2019 8 11 1873 31694225 10.3390/jcm8111873 PMC6912689

[20] Şahin M M Yılmaz M Karamert R Te evaluation of Caldwell–Luc operation in the endoscopic era; experience of the last 7 years J Oral Maxillofac Surg 2020 78 09 1478 1483 32464104 10.1016/j.joms.2020.04.024

[21] Lee F C Fernandes C MC Murrell H C Classification of the maxillary sinus according to area of the medial antral wall: a comparison of two ethnic groups J Maxillofac Oral Surg 2009 8 02 103 107 23139485 10.1007/s12663-009-0027-6 PMC3453934

[22] Wagner F Dvorak G Nemec S Morphometric analysis of sinus depth in the posterior maxilla and proposal of a novel classification Sci Rep 2017 7 45397 28338085 10.1038/srep45397 PMC5364414

